# Immune Modulation by Different Types of β2→1-Fructans Is Toll-Like Receptor Dependent

**DOI:** 10.1371/journal.pone.0068367

**Published:** 2013-07-05

**Authors:** Leonie Vogt, Uttara Ramasamy, Diederick Meyer, Gerdie Pullens, Koen Venema, Marijke M. Faas, Henk A. Schols, Paul de Vos

**Affiliations:** 1 Department of Pathology and Medical Biology, University Medical Center Groningen, University of Groningen, Groningen, The Netherlands; 2 Laboratory of Food Chemistry, Wageningen University, Wageningen, The Netherlands; 3 Scientific and Regulatory Affairs, Sensus B.V., Roosendaal, The Netherlands; 4 Product Development, Cosun Food Technology Centre, Roosendaal, The Netherlands; 5 Department of Biosciences, TNO, Zeist, The Netherlands; Charité, Campus Benjamin Franklin, Germany

## Abstract

**Introduction:**

β2→1-fructans are dietary fibers. Main objectives of this study were 1) to demonstrate direct signalling of β2→1-fructans on immune cells, 2) to study whether this is mediated by the pattern recognition receptors Toll-like receptors (TLRs) and nucleotide-binding oligomerisation domain-containing proteins (NODs), and 3) to relate the observed effects to the chain length differences in β2→1-fructans.

**Methods:**

Four different β2→1-fructan formulations were characterised for their chain length profile. Human peripheral blood mononuclear cells (PBMCs) were stimulated in vitro with β2→1-fructans, and production of IL-1Ra, IL-1β, IL-6, IL-10, IL-12p70, and TNF-α was analysed. Reporter cells for TLRs and NODs were incubated with β2→1-fructans and analysed for NF-κB/AP-1 activation.

**Results:**

Cytokine production in human PBMCs was dose- and chain length-dependent. Strikingly, short chain enriched β2→1-fructans induced a regulatory cytokine balance compared to long chain enriched β2→1-fructans as measured by IL-10/IL-12 ratios. Activation of reporter cells showed that signalling was highly dependent on TLRs and their adapter, myeloid differentiation primary response protein 88 (MyD88). In human embryonic kidney reporter cells, TLR2 was prominently activated, while TLR4, 5, 7, 8, and NOD2 were mildly activated.

**Conclusions:**

β2→1-fructans possess direct signalling capacity on human immune cells. By activating primarily TLR2, and to a lesser extent TLR4, 5, 7, 8, and NOD2, β2→1-fructan stimulation results in NF-κB/AP-1 activation. Chain length of β2→1-fructans is important for the induced activation pattern and IL-10/IL-12 ratios.

## Introduction

High fiber intake is associated with lower mortality in subjects suffering from circulatory, digestive, and non-cardiovascular non-cancerous inflammatory diseases [1]-[3]. These associations are similar for men and women and are observed in most countries even after careful adjustment for potentially confounding lifestyle and dietary differences [4]. The mechanisms by which fibers contribute to reduced mortality remain to be identified but have been suggested to be of both metabolic and immunological nature [5]. Cereal fibers have been associated with lower concentrations of inflammatory biomarkers [4], but there is an urgent need for studies addressing specific fibers [4] to obtain a better understanding of mechanisms and components underlying protective associations.

One of the many types of dietary fibers which have been reported to elicit health benefits are linear β2→1-fructans (also named inulin-type fructans, ITFs, or fructooligosaccharides). These fructans are made up of fructose subunits, can vary considerably in chain length, and occur with or without the presence of a terminal glucose moiety [6]. The fructose oligomers are denoted as F_n_ or GF_n_ (_n_ = number of fructose subunits, GF_n_ = fructose chain terminated with a glucose molecule) [6]. The chain length can also be denoted by the Degree of Polymerisation (DP), representing the number of monosaccharide subunits in the chain [6]. Whether these variations in composition cause different physiological responses is currently unknown. Ingestion of β2→1-fructans induces specific effects on the immune system, as recently reviewed [7]-[11]. Changes in immunological parameters have been reported in the gut lumen, Peyer’s patches, spleen, and blood [12]-[14]. These effects could be induced via a prebiotic mechanism by stimulating beneficial (“probiotic”) bacteria in the intestine, such as bifidobacteria and lactobacilli [10]. This can coincide with modulation of fermentation products such as increased production of short chain fatty acids [15], [16], which affect the recruitment of leukocytes to inflammatory sites [17], [18], and suppress the production of pro-inflammatory cytokines and chemokines [18]-[20]. Besides through these more indirect effects on the microbiota and its fermentation products, another pathway of modulation is gaining attention, i.e. through direct signalling on immune cells. This theory is supported by new insights into the structural and functional makeup of the intestine [21], [22]. Some dietary fibers such as β-glucans, modulate the human immune system by binding to pattern recognition receptors (PRRs) on cells of the innate immune system, such as Dectin-1 and Toll-like receptors (TLRs) [23]-[25]. These PRRs recognise so-called pathogen-associated molecular patterns (PAMPs), which are small molecular motifs found on groups of pathogens but also on many microorganisms with beneficial or no effects on host health [26]. Because of their ability to recognise specific carbohydrate moieties and elicit immune responses, we hypothesised that PRRs, and more specifically TLRs and NODs are activated by β2→1-fructans, and that this is one of the pathways by which β2→1-fructans can influence the human immune system [27], [28].

As little knowledge is available on the specific immunomodulating effects of different types of β2→1-fructans, we investigated the effect of β2→1-fructans with different chain lengths on cytokine release by human Peripheral Blood Mononuclear Cells (PBMCs) and we studied whether and which PRRs are involved in β2→1-fructan recognition. Our studies support the concept that ITFs can signal directly on immune cells via TLRs and that chain length differences in ITFs can differentially affect immunological parameters.

## Materials and Methods

### Ethics Statement

Chemical analyses (HPAEC and HPSEC) were performed at Wageningen University. Other experiments, including blood sampling of human volunteers, were conducted within the University Medical Center Groningen, in the Netherlands. Written informed consent was obtained, and data were analysed and presented anonymously. This research and consent procedure have been approved by the ethical review board of the University Medical Center, Medisch Ethische Toetsingscommissie University Medical Center Groningen, as documented in the approved application “2007/255”. All clinical investigation was conducted according to the principles expressed in the Declaration of Helsinki.

### High Performance Anion Exchange Chromatography

Four different β2→1-fructan formulations were selected and their specific chain length profiles (range and distribution) were characterised by High Performance Anion Exchange Chromatography (HPAEC). The applied β2→1-fructans were extracted from chicory root and comprise a mixture of different chain lengths varying from DP 2 to 60, referred to as inulin or native inulin [29]. These chicory root β2→1-fructan chains are terminated by a glucose molecule [6]. Fructans of varying DP can be acquired by partial enzymatic hydrolysis of this extract resulting in GF_n_ and F_n_ chains with GF_n_ ranging from DP 2 to 10 [6]. Another way to obtain β2→1-fructans is to synthesise them from sucrose, resulting in GF_n_ type fructans with DP 2-4 [30].

The following β2→1-fructan formulations were analysed: ITF I (Frutalose©OFP) ITF II (Frutafit©CLR), ITF III (Frutafit©IQ), and ITF IV (Frutafit©TEX!). β2→1-fructan formulations were provided by Sensus B.V., Roosendaal, The Netherlands. Endotoxin levels (endotoxin units, EU) of all used β2→1-fructan samples were assessed by Toxikon (Leuven, Belgium) and were below 0.3 x 10^-3^ µg^-1^.

For HPAEC, a Dionex ICS 3000 system (Dionex), equipped with a Dionex CarboPac PA-1 column (2 x 250 mm) in combination with a CarboPac PA-1 guard column (2 x 50 mm) was used. Sample concentrations of 0.05–0.1 mg/ml and partial-loop injection of 10 µl were applied. The system was equipped with pulsed amperometric detection. Elution was performed at 0.3 ml/min. and oligomers were separated using a gradient as follows: 0-400mM NaOAc in 100 mM NaOH within 40 min., followed by a 5 min. washing step (1M NaOAc in 100 mM NaOH), and 15 min. equilibration (100 mM NaOH).

### High Pressure Size Exclusion Chromatography

β2→1-fructan formulations were tested for their elution profile using High Performance Size Exclusion Chromatography (HPSEC) on Dionex Ultimate 3000 HPLC (Dionex, Sunnyvale, CA, USA). The analysis was performed on three TSK-Gel columns connected in series (4000-3000-2500 SuperAW; 150 x 6 mm). The columns were preceded by a TSK Super AW-L guard column (35 x 4.6 mm). All columns were from Tosoh Bioscience (Tokyo, Japan). Elution was performed at a flow rate of 0.6 ml/min. using sodium nitrate (0.2 M) as the eluent. A volume of 20 µl of the sample (2.5 mg/ml) in millipore water was injected and eluted at 55°C. Solubles were detected using a refractive index detector, Shodex type RI 101 (Showa Denko, Japan). The software used for acquiring the data was Chromeleon version 7. Molecular weight distribution of polysaccharides was determined using pullulan standards (Polymer Laboratories, Varian Inc., Palo Alto, CA, USA) in the molecular mass range of 0.18 - 790 kDa.

### Isolation Human Peripheral Blood Mononuclear Cells

Human PBMCs were used to study whether β2→1-fructans can signal on immune cells, especially since PBMCs express many PRRs such as TLRs [31], [32] and NODs [33]. Peripheral blood from human volunteers was collected in heparinised tubes (15 IU/ml lithium-heparin, Becton Dickinson B.V., Breda, The Netherlands) and PBMCs were isolated by Ficoll density gradient separation (Lymphoprep, Axis-Shield, Oslo, Norway). Cells were kept in RPMI1640 medium (Gibco, Life Technologies, Bleiswijk, The Netherlands) supplemented with 10% Fetal Bovine Serum (FBS, HyClone, Thermo Scientific, Breda, The Netherlands) and 50 µg/ml gentamicin (Gibco).

### Cytokine Expression

As a read out for PBMC activation, cytokine production in the medium was analysed after stimulation of PBMCs with a concentration series of β2→1-fructans. To evaluate whether β2→1-fructans induce a more anti-inflammatory or pro-inflammatory effect, and whether chain length profile would affect this balance, the IL-10/IL-12 ratio was calculated for each β2→1-fructan formulation at 1 µg/ml or 100 µg/ml. In this context, a higher IL-10/IL-12 ratio is representative of a more anti-inflammatory effect [34], [35]. Human PBMCs (n = 6; 3 males, 3 females, age 25-42 yr., healthy non-smoker volunteers) were seeded in a 24 wells plate at a density of 2×10^6^ cells/well with a final volume of 1 ml/well. To characterise possible effects of different chain length profiles on cytokine expression, the β2→1-fructan formulations ITF I to IV were dissolved in culture medium and added to the PBMCs at final concentrations ranging from 1 to 100 µg/ml. After 24h of incubation (37°C, 5% CO_2_) cytokine levels in the supernatant were measured using a Bio-Plex™ premixed cytokine assay, human 6-plex group I; cat.#: M5000B6CPS, control 5022016, according to the manufacturer’s instructions. (Bio-rad Laboratories, Veenendaal, The Netherlands). This customised kit simultaneously measured human IL-1Ra, IL-1β, IL-6, IL-10, IL-12p70, and TNF-α. Concentration series of cytokine standards were prepared for the appropriate concentration range, and coupled beads were diluted ten times, resuspended, and added to a pre-wetted filter plate. After washing the plate twice, standards, negative controls, and samples (all in duplicate) were transferred into the plate (50 µl per well), and the plate was sealed and incubated on a shaker at room temperature (RT) for 30 min. After incubation, the plate was washed three times, detection antibodies were resuspended and diluted ten times and 25 µl was added to each well. The plate was incubated on a shaker at RT for 30 min., and after washing three times, 50 µl of streptavidin-phycoerythrin was added to each well and the plate was incubated on a shaker at RT for 10 min. After washing the plate three times, 125 µl of assay buffer was added per well, the plate was incubated on a shaker for 5 min. and fluorescence was measured using a Luminex 100 System and StarStation software. All procedures as of the incubation with detection antibodies were performed in the dark.

### Cell Culture of Reporter Cell Lines

Selection media, Normocin antibiotic, Quanti-blue reagent, TLR agonists and the following TLR-, and NOD-reporter cell lines were acquired from InvivoGen (InvivoGen, Toulouse, France). Two THP-1 human acute monocytic leukemia reporter cell lines were acquired from Invivogen, both endogenously expressing human TLRs and with inserted construct for Secreted Embryonic Alkaline Phosphatase (SEAP) coupled to the NF-κB/AP-1 promoter. The first of these THP-1 cell lines carries extra inserts for MD2 and CD14 to boost TLR signalling, and the second THP-1 cell line expresses only a truncated, non-functional form of the TLR adapter MyD88. Nine different human Embryonic Kidney (HEK293)-Blue reporter cell lines were purchased from Invivogen, each with a different inserted construct for either human TLR2, TLR3, TLR4, TLR5, TLR7, TLR8, TLR9, NOD1, or NOD2, and all nine cell lines carrying an inserted construct for Secreted Embryonic Alkaline Phosphatase (SEAP) coupled to the NF-κB/AP-1 promoter. Both THP-1 cell lines were maintained in RPMI1640 containing 10% heat inactivated FBS, NaHCO_3_ (Boom B.V. Meppel, The Netherlands; 1,5 g/l), L-glutamine (2 mM), glucose (4,5 g/l), HEPES (10 mM), sodium pyruvate (1 mM) penicillin/streptomycin (50 U/ml and 50 µg/ml), all from Sigma-Aldrich Chemie B.V., Zwijndrecht, The Netherlands, and Normocin (100 µg/ml). Both THP-1 cell lines were kept at a concentration of 5×10^5^ cells/ml. HEK-Blue cells were maintained in DMEM (Life Technologies Europe B.V.) containing 10% heat inactivated FBS, L-glutamine (2 mM), glucose 4,5 g/l), penicillin/streptomycin (50 U/ml and 50 µg/ml), and Normocin (100 µg/ml). HEK cells were grown to ∼80% confluency. After culturing for 3 passages, all reporter cell lines were maintained in selection media according to the manufacturer’s protocol.

### Reporter Cell Stimulations and Quanti-Blue™ Analysis

THP-1 cells were centrifuged for 5 min. at 300 *g* to collect the cells and resuspended to the cell density specified by the manufacturer’s protocol (Table 1). In a flat bottom 96 wells plate, 100 µl of this cell suspension per well was stimulated for 24 h (37°C, 5% CO_2_) with 10 µl of stimulus, i.e. concentration series (1 µg/ml - 2 mg/ml) of β2→1-fructan formulations ITF I to IV, or the relevant positive control as indicated by the manufacturer’s protocol (Table 1). Plain culture medium and endotoxin-free water were applied as negative controls. After incubation, 20 µl of cell culture medium of stimulated reporter cells was incubated with 180 µl of Quanti-blue reagent in a new flat bottom 96 wells plate for 45 min at 37°C and SEAP activity (absorbance), representing activation of NF-κB/AP-1, was measured at 650 nm on a VersaMax microplate reader (Molecular Devices GmbH, Biberach an der Riss, Germany) using SoftMax Pro Data Acquisition & Analysis Software. HEK-Blue reporter cells were rinsed with medium to detach them from the culture flask and cells were resuspended to the cell density specified by the manufacturer’s protocol (Table 1). 180 µl of cell suspension per well was stimulated for 24 h (37°C, 5% CO_2_) with 20 µl of β2→1-fructan, endotoxin free water, plain culture medium or positive control (Table 1) in a flat bottom 96 wells plate. For all HEK cell lines, after incubation, analysis of SEAP was performed in the same way as described for the THP-1 cells.

**Table 1:** Cell densities and positive controls used in reporter cell stimulations.

Cell line	Cell density	Positive control
THP-1 MD2-CD14	1*10^6^ cells/ml	*E.coli* K12 lipopolysaccharide Ultrapure (LPS, 100 ng/ml)
THP-1 DefMyD	2*10^6^ cells/ml	L-Ala-gamma-D-Glu-mDAP (TriDAP, 10 µg/ml)
HEK-Blue TLR2	2.8*10^5^ cells/ml	Pam2CGDPKHPKSF (FSL-1, 1 µg/ml)
HEK-Blue TLR3	2.8*10^5^ cells/ml	Poly(I:C) high molecular weight (HMW, 1 mg/ml)
HEK-Blue TLR4	1.4*10^6^ cells/ml	*E.coli* K12 lipopolysaccharide Ultrapure (LPS, 100 ng/ml)
HEK-Blue TLR5	1.4*10^6^ cells/ml	Recombinant flagellin from *S.typhimurium* (Rec-FLA-ST,100 ng/ml)
HEK-Blue TLR7	2.2*10^6^ cells/ml	Imiquimod (5 µg/ml)
HEK-Blue TLR8	2.2*10^6^ cells/ml	Single stranded RNA (ssRNA40/LyoVec™, 5 µg/ml)
HEK-Blue TLR9	4.5*10^6^ cells/ml	Type B CpG oligonucleotide (ODN 2006, 100 mg/ml)
HEK-Blue NOD1	2.8*10^6^ cells/ml	L-Ala-gamma-D-Glu-mDAP (TriDAP, 10 µg/ml)
HEK-Blue NOD2	1.4*10^6^ cells/ml	MurNac-L-Ala-gamma-D-Glu-mDAP (M-TriDAP, 10 µg/ml)

### Statistical Analysis

Significance levels were determined by parametric Student’s t-test for unpaired observations (two-tailed) or by non-parametric Mann-Whitney U-test for unpaired observations (two-tailed). Results are expressed as mean ± SEM or mean ± SD respectively. A *P*-value <0.05 was considered statistically significant. *P-*values <0.05 are denoted with *, *P*-values <0.01 are denoted with **, and *P*-values <0.001 are denoted with ***.

## Results

### Chemical Characterisation of β2→1-fructan Formulations

**Figure 1:**
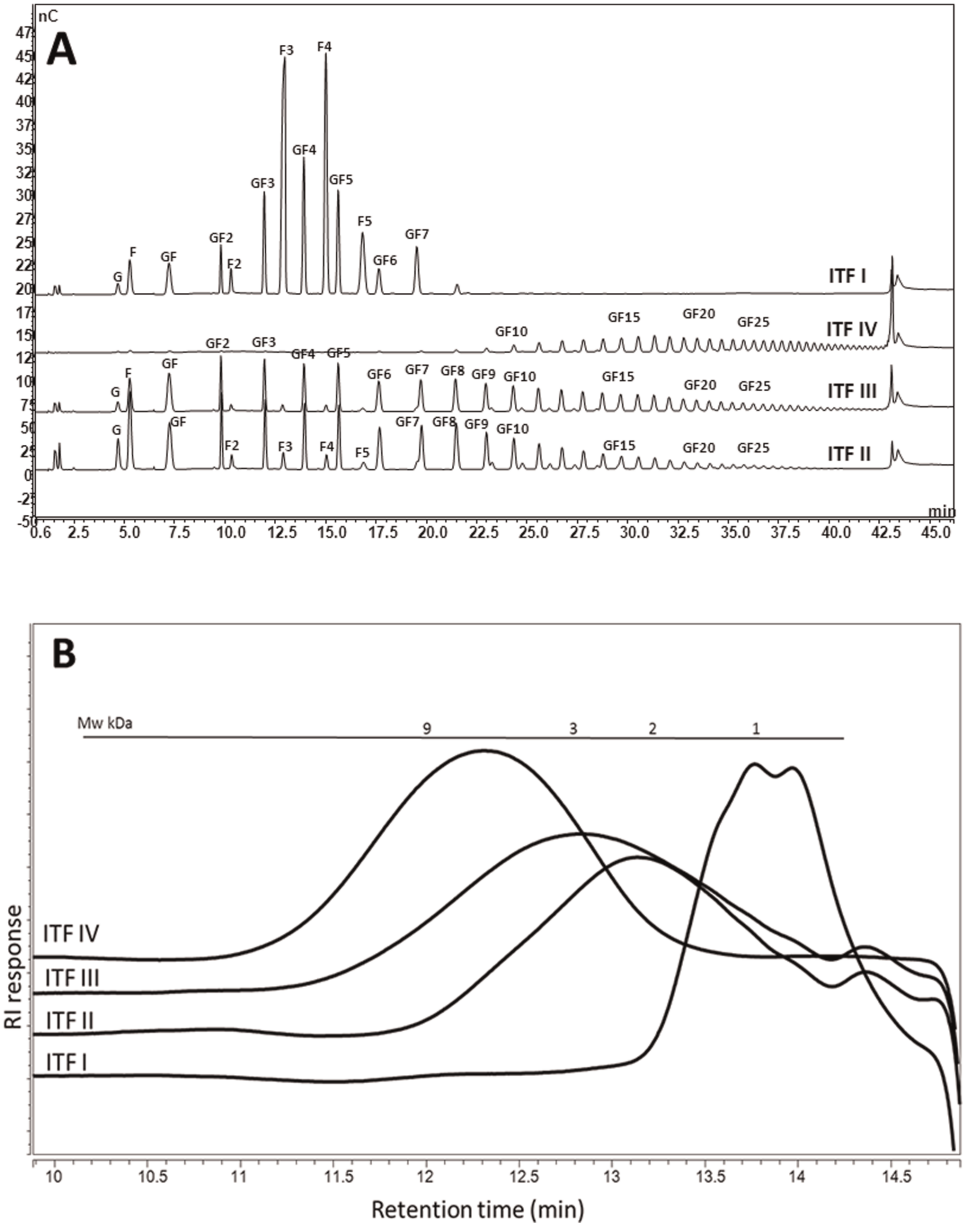
HPAEC profiles and HPSEC elution patterns of different β2→1-fructan formulations. Figure A depicts the fructose (F) and glucose (G) monomers, dimers, and fructan oligomers present in the ITF formulations. GF_n_ chains are terminated by a glucose molecule, and F_n_ chains consist of only fructose moieties. In both cases, n represents the number of fructose moieties in the chain. Figure B depicts the elution patterns as a measure for molecular weight distribution profiles of the four different β2→1-fructan formulations in kDa.

The oligomer profiles of four β2→1-fructan formulations were characterised using HPAEC and HPSEC. Figure 1A depicts the oligosaccharide range and relative response per oligomer of the β2→1-fructan formulations tested with HPAEC. Figure 1B represents the HPSEC analysis of the four different β2→1-fructan formulations, showing the elution patterns as a measure for the molecular size distributions of these compounds in kDa. The HPSEC analysis corroborated the observed chain length profiles from the HPAEC analysis and in addition this method rendered a visual representation of the degree in which the different chain lengths are present per analysed formulation. The most important differences can be observed between ITF I and II as compared to ITF III and IV. ITF I can described as fructooligosaccharide (FOS) with mostly chain lengths of DP<10, and ITF II can be described as a FOS-enriched inulin, with a large proportion of chains smaller than DP10, but also containing chains with DP up to 25. ITF III and IV are described as “inulin” due to their broad range of chain lengths present (up to DP60). ITF I and II contain mostly chains of the type GF_3_, GF_4_, and GF_5_, (i.e. starting with a glucose molecule followed by 3, 4, or 5 fructose subunits) and F_3_, F_4_, or F_5_ (chains consisting only of 3, 4, or 5 fructose moieties). Between ITF I and II, ITF I contains relatively more F_n_ type oligosaccharides and ITF II contains relatively more GF_n_ type molecules. ITF III and IV consist solely of the GF_n_ type fructans while ITF I and II consist of both GF_n_ and F_n_ fructans. Glucose and fructose monomers, and GF and GF_2_ (i.e. dimers of glucose and fructose subunits and trimers made up of one glucose subunit and two fructose subunits) are present in all four ITFs.

### β2→1-fructans Induce Cytokine Production in Human PBMCs

**Figure 2:**
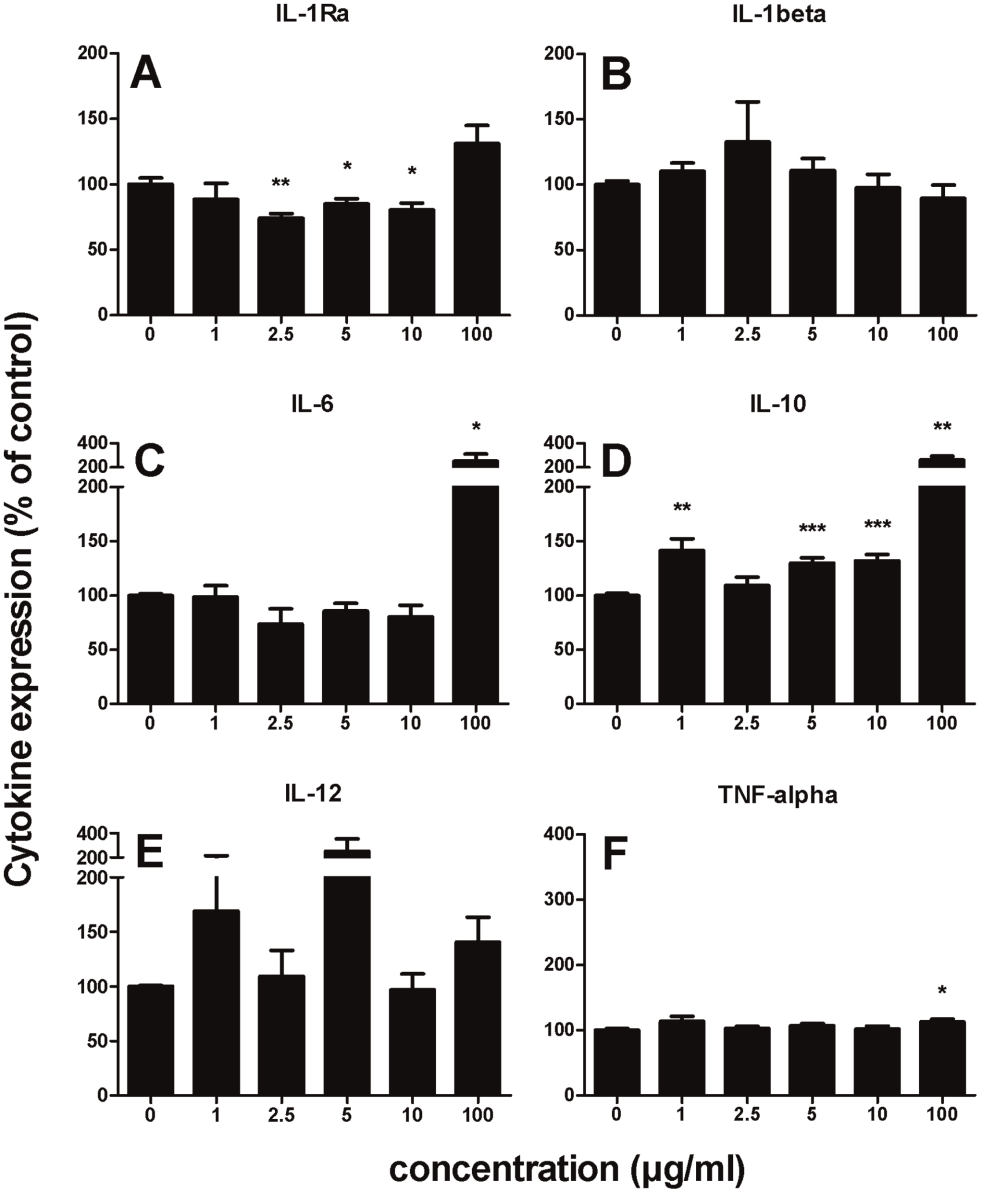
Induction of cytokines by ITF I, in a dose range of 0 to 100 µg/ml. Statistical significance levels were determined with a parametric Student’s t-test for unpaired observations (two-tailed). Mean and SEM of cytokine production is plotted as percentage of controls, which were set to 100% (n = 4). Panels A to F show the results for IL-1Ra, IL-1β, IL-6, IL-10, IL-12, and TNF-α respectively.

To gain insight into dose effects and to determine which cytokines are induced by β2→1-fructans we first screened for a panel of pro- and anti-inflammatory cytokines, i.e. IL-1β, IL-6, IL-12, TNF-α, IL-1Ra, and IL-10, after stimulating human PBMCs for 24 hours with β2→1-fructans. This was done for ITF I, in doses of 1/2,5/5/10, and 100 µg/ml. As shown in Figure 2, IL-1Ra expression was decreased for lower doses (2,5 to 10 µg/ml β2→1-fructans) and increased at 100 µg/ml β2→1-fructans (p<0.05, panel A). The same pattern was observed for IL-6; expression was decreased for 2.5 µg/ml (p<0.05) and a substantial increase was observed at 100 µg/ml (p<0.01, panel C). IL-10 expression was increased, especially at 1 µg/ml (p<0.001) and 100 µg/ml (p<0.0001, panel D). A small increase for TNFα was observed at 100 µg/ml (12%, p<0.05, panel F). Since a dose as low as 1 µg/ml induced a significant increase for IL-10, and 100 µg/ml induced significant and substantial increases, these low end and high end doses were subsequently used for further cytokine measurements in PBMCs.

### Inulin-type Fructan-induced Cytokine Production by Human PBMCs is Chain Length Dependent

**Figure 3:**
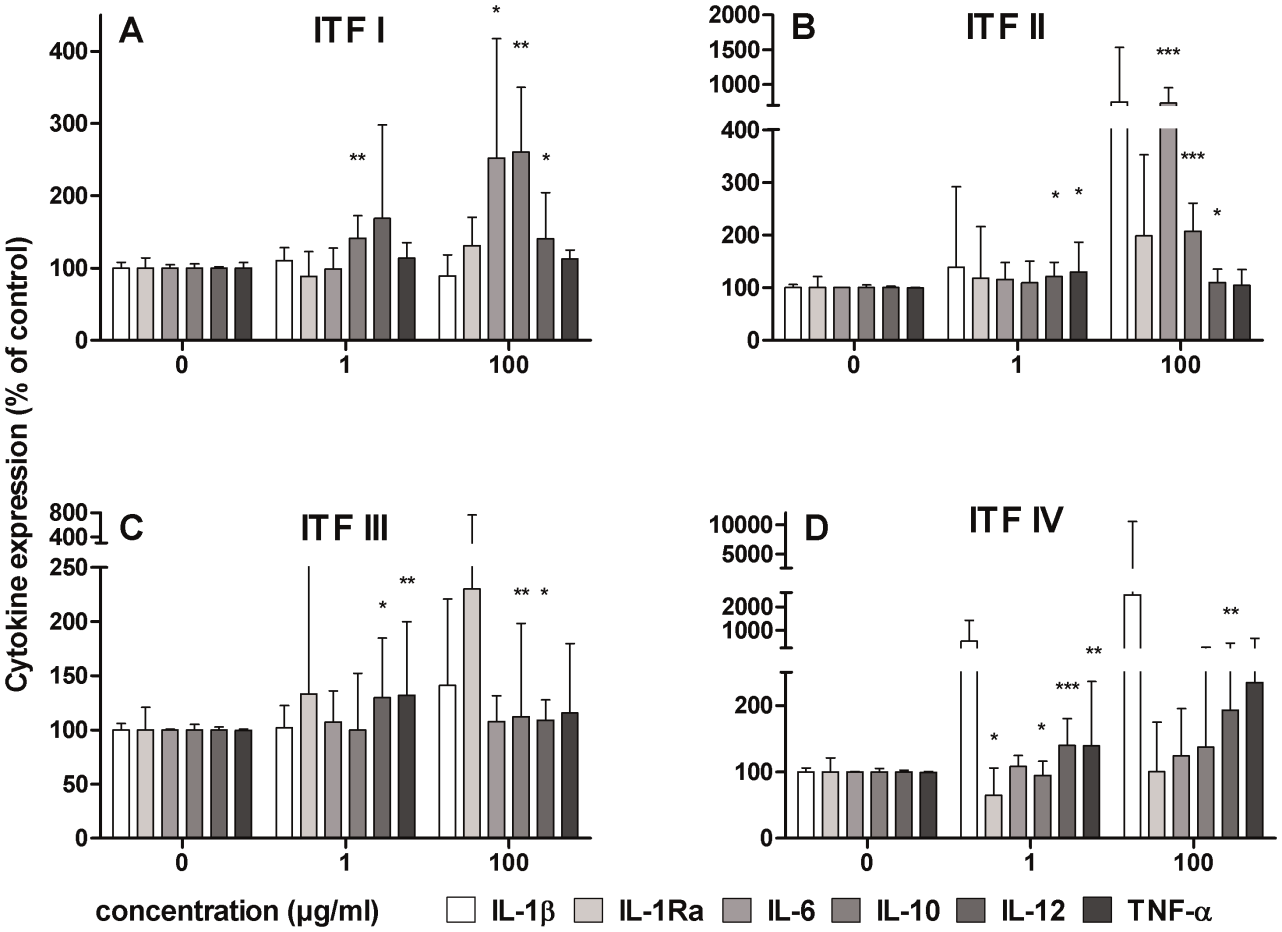
Induction of cytokines by β2→1-fructan formulations with different chain length (DP range), at 0, 1, and 100 µg/ml. Statistical significance levels were determined with a non-parametric Mann-Whitney U-test for unpaired observations (two-tailed). Mean and SD of cytokine expression are plotted as percentage of controls (represented by 0 µg/ml), which were set to 100% (n = 6). Panel A to D represent cytokine expression induced by ITF I to IV respectively.

To gain insight into size-response relationships, we investigated whether the chain length of β2→1-fructans has an effect on the type and quantity of cytokines produced by PBMCs. ITF I to IV were tested at the concentrations of 1 and 100 µg/ml. IL-6 and IL-10 were mainly induced by ITF I and ITF II (Fig. 3) which can therefore be mainly attributed to short chain molecules enriched in these formulations, i.e. F_2_-F_5_ and GF_2_-GF_5_ respectively. Production of IL-12 was slightly increased by ITF II and strongly induced by ITF III and IV, indicating that longer chains (>DP8, GF_n_ type) have to be held responsible for this effect. Similarly, the highest production of TNF-α was observed after incubation with ITF IV. These combined results showed that chain length profile of β2→1-fructan formulations is an important determinant of the cytokine profile which is induced upon stimulation.

**Figure 4:**
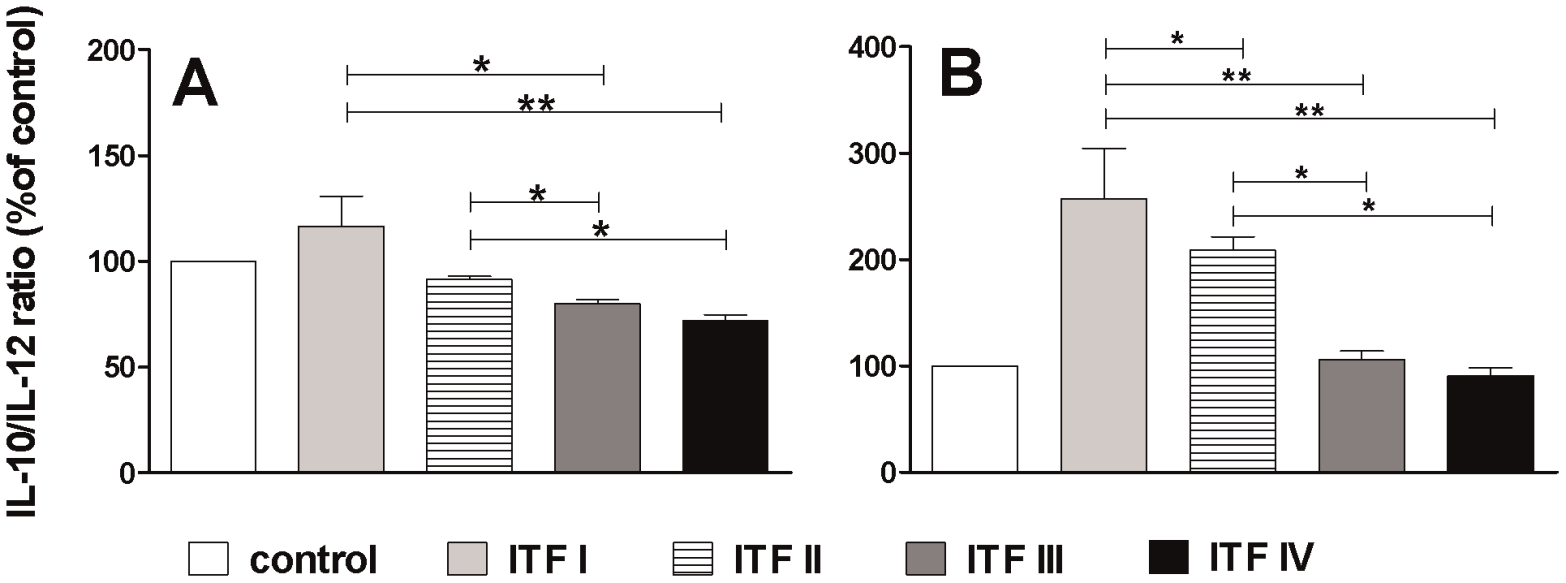
Ratio of IL-10/IL-12 upon incubation of PBMCs with different chain length β2→1-fructans. PBMCs (n = 6) were stimulated with 1 µg/ml (panel A) and 100 µg/ml (panel B) β2→1-fructans for 24h. Statistical significance levels were determined with a non-parametric Mann-Whitney U-test for unpaired observations (two-tailed). Mean and SD of the IL-10/IL-12 ratios is plotted for the different β2→1-fructans as percentage of controls, which were set to 100% (n = 6) and horizontal bars indicate the significant differences between β2→1-fructan treatments.

Next, the IL-10/IL-12 ratios were calculated to quantify whether there is a correlation between chain length profile and cytokine balance (Fig.4). IL-10/IL12 ratios can be applied to calculate the regulatory effects of bioactive food components [36]. Controls were set to 100, and a ratio of more than 100 was considered to be regulatory or anti-inflammatory, whereas a ratio lower than 100 was considered to be proinflammatory. Strikingly, the IL-10/IL-12 ratios gradually decreased in the sequence of ITF I>ITF II>ITF III>ITF IV, indicating that shorter chain fructans induce a regulatory cytokine balance in human PBMCs compared to longer chain fructans. When taking the structural differences into consideration (Fig.1) it can be concluded that the molecules F_2_-F_5_ and/or GF_2_-GF_5_, which are enriched in ITF I and II, skew the IL-10/IL-12 ratio in PBMCs more towards IL-10, and thus induce a more anti-inflammatory balance.

### TLR Mediated Activation of NF-κB/AP-1 by β2→1-fructans is Dependent on the Presence of Functional TLR Adapter MyD88

Next, we investigated whether the activation of immune cells can be explained by activation of PRRs by the β2→1-fructans. To this end we applied the following strategy. First, β2→1-fructans were tested for the ability to induce NF-κB/AP-1 transcription in a THP-1 reporter cell line which endogenously expresses all TLRs, and carries extra inserts for co-signalling molecules CD14 and MD2 to increase the TLR-mediated responses. NF-κB and AP-1 are essential transcription factors in signalling for cytokine release [37].

**Figure 5:**
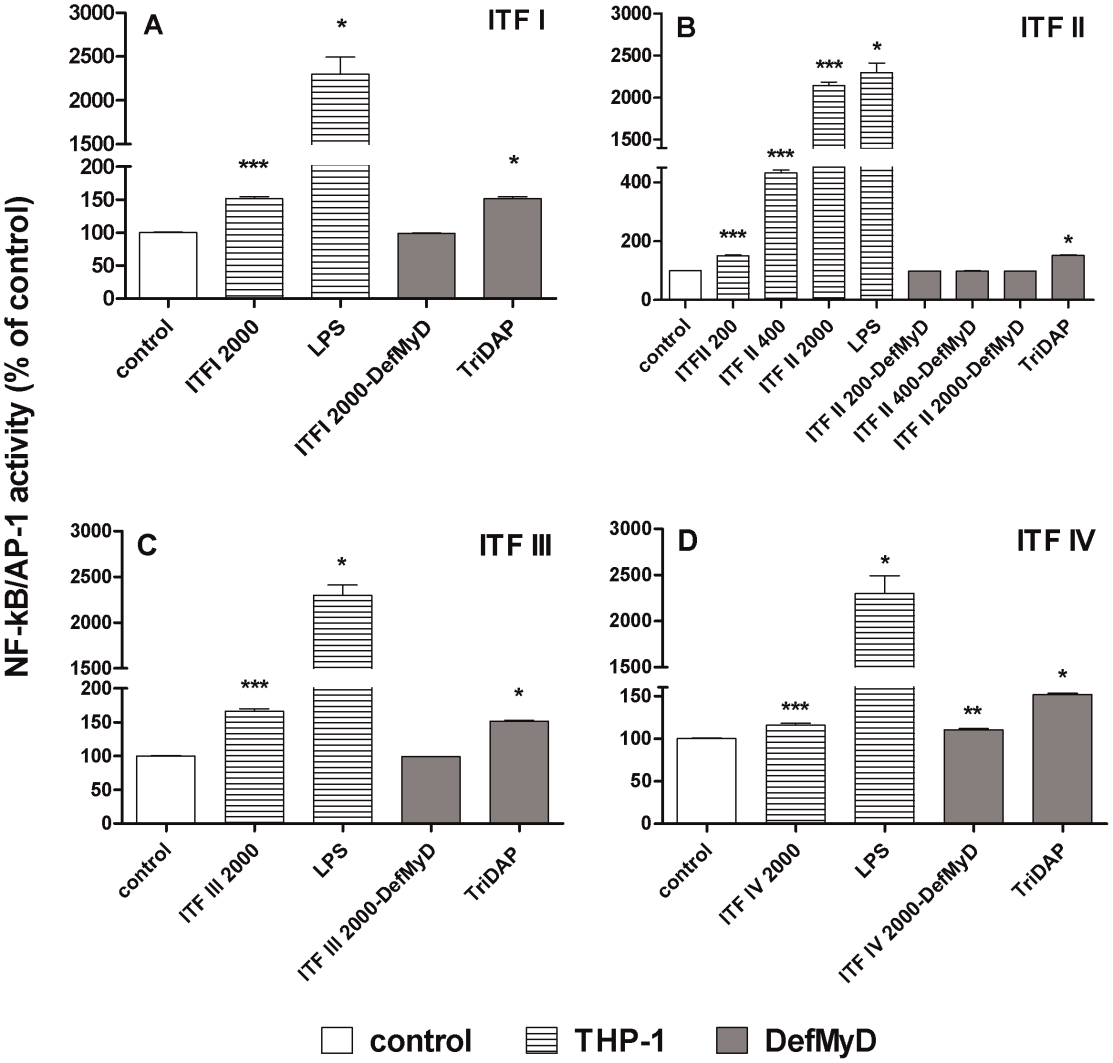
NF-κB/AP-1 activation in THP-1 MD2-CD14 and THP-1 defMyD reporter cells. Statistical significance levels were determined with a non-parametric Mann-Whitney U-test for unpaired observations (two-tailed). Mean and SD of NF-κB/AP-1 activation by β2→1-fructans of different chain lengths (ITF I, II, III and IV) are plotted as percentage of negative controls (unstimulated cells), which were set to 100% for both cell lines. LPS stimulation was used as a common positive control for TLR4/MyD88 signalling to NF-κB/AP-1 in the functional THP-1 MD2-CD14 cell line. TriDAP was used as a positive control in THP-1 DefMyD cells, which induces MyD88-independent signalling to NF-κB/AP-1. Panel A to D represent cytokine expression induced by ITF I to IV respectively. Only concentrations which induced activation in the THP-1 MD1-CD14 cells are shown for comparison of these concentrations in the THP-1 DefMyD cells.

ITF I and II induced a statistically significant elevation of NF-κB/AP-1 activation in THP-1 MD2-CD14 cells, while ITF III and ITF IV only induced activation at higher doses (Fig.5). The strongest activation was observed for ITF II, which is mainly short chain GF_n_. Next, we investigated whether the activation was TLR-dependent by testing the effect of β2→1-fructans on a specific THP-1 reporter cell line carrying the truncated TLR adapter molecule MyD88. These THP-1 DefMyD cells express only non-functional MyD88, whereas the functional MyD88 is an essential adapter molecule for TLR2, 4, 5, 7, 8, and 9 signalling. The β2→1-fructan doses which significantly activated THP-1 MD2-CD14 cells were subsequently tested in the THP-1 DefMyD cells.

β2→1-fructan mediated NF-κB/AP-1 activation appeared to be TLR dependent as the activation pattern as observed in THP-1 MD2-CD14, was virtually absent in the MyD88 deficient cell line. This indicated that β2→1-fructan mediated signalling to NF-κB/AP-1 was TLR2, 4, 5, 7, 8, and/or 9 dependent. Slight activation of THP-1 DefMyD cells by ITF IV suggests that other activation pathways independent of MyD88 may also have been induced by high concentrations of the longer chain β2→1-fructans which are enriched in this ITF.

### β2→1-fructans Induce Strong Dose Dependent Activation of TLR2, Slight Activation of TLR4, 5, 7, 8, and NOD2, but No Activation of TLR3, TLR9, or NOD1

**Figure 6:**
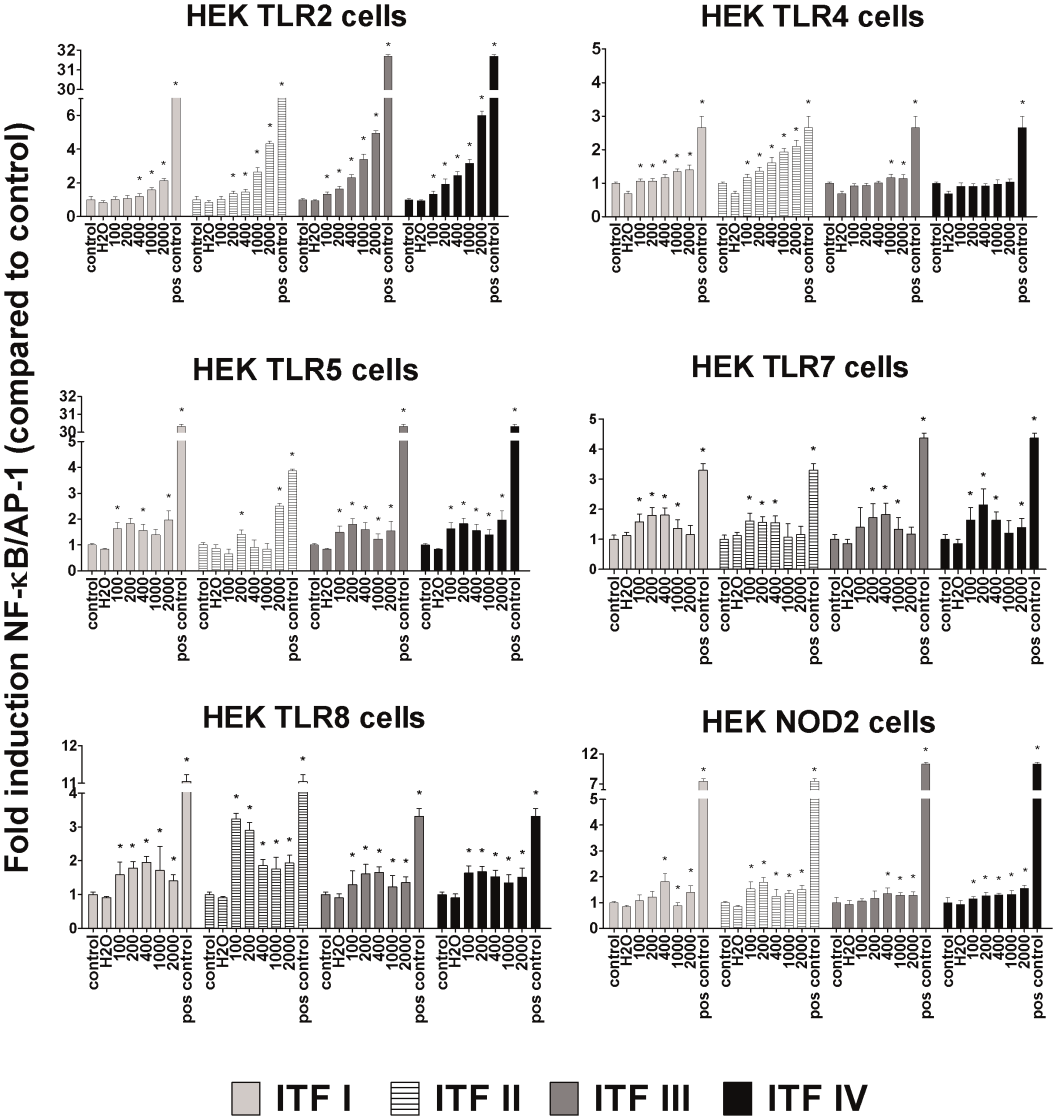
NF-κB/AP-1 activation of HEK cell lines overexpressing separate TLRs or NODs. Statistical significance levels were determined with a non-parametric Mann-Whitney U-test for unpaired observations (two-tailed). Mean and SD of NF-κB/AP-1 activation in HEK cell lines stimulated with β2→1-fructans for 24 h are plotted as percentage of unstimulated controls, which were set to 100%. Dosages are plotted in µg/ml. Endotoxin free H_2_O was used as an additional negative control and per cell line the relevant positive controls were applied as mentioned in Table 1.

The results from the THP-1 cell stimulations showed that TLRs are involved in β2→1-fructan signalling. Next, we investigated which TLRs are specifically activated by β2→1-fructans and whether the intracellular PRRs NODs were activated. To this end, HEK reporter cell lines, each carrying one construct for a specific TLR or NOD, were stimulated with β2→1-fructans in the same way as described for the THP-1 cells (Fig.6). Strikingly, HEK cells with TLR2 construct were strongly and dose dependently activated by the β2→1-fructans. Moreover, this response was chain length dependent, as NF-κB/AP-1 activation in HEK TLR2 cells increased with increasing chain length in the sequence of ITF I<ITF II<ITF III<ITF IV. In these cells, long chain fructans induced the strongest activation, up to 6-fold induction as compared to control. To a lesser extent, HEK cells carrying either TLR4, 5, 7, 8, or NOD2 were activated upon β2→1-fructan stimulation. HEK cells expressing TLR3, 9 or NOD1 were not significantly activated by β2→1-fructan stimulation (data not shown). These data combined show that TLR binding was β2→1-fructan type-, and thus chain length dependent.

## Discussion

We hypothesised that β2→1-fructans can affect the immune status through physical contact with pattern recognition receptors (PRRs) on gut immune cells such as intra-epithelial lymphocytes [38] or DCs [27], [28]. As TLR activation in PBMCs leads to cytokine production [31], [39], we used these cells to test whether β2→1-fructans would also induce production of cytokines. Both anti-inflammatory and proinflammatory cytokines were produced, demonstrating that β2→1-fructans have the ability to activate human immune cells. Interestingly, chain length proved to be an important factor in skewing the cytokine balance. Anti-inflammatory IL-10 was strongly induced by short chain β2→1-fructans and a striking correlation was observed between the ratio of IL-10/IL-12 and the chain lengths of the fibers. The IL-10/IL-12 ratio has previously been used to evaluate the effects of probiotic bacteria on immune responses [40], [41], and to describe anti-inflammatory effects of butyrate [42]. It is therefore useful to apply the same approach to study the effect of prebiotic fibers.

It can be concluded on the basis of our data that the short chain molecules which are more abundant in ITF I and II, skew the IL-10/IL-12 ratio in PBMCs more towards IL-10, and thus induce a more anti-inflammatory balance. When considering that the proportion of short chain fructans of the GFn type are also distributed as ITF I>ITF II>ITF III, the results also suggest that specifically the short chain fructans of the GF_n_ type determine the outcome of this cytokine ratio. In spite of the fact that longer chains induced more IL-12 compared to shorter chains, the induced IL-10/IL-12 ratio is not significantly different from the ratio measured in the control cells, indicating that they are pro-inflammatory as compared to the shorter chains but not pro-inflammatory per se, when compared to controls.

IL-6 production was observed for higher concentrations of ITF I and II. Although IL-6 production is generally regarded as proinflammatory, IL-6 can exert several anti-inflammatory effects such as inhibition of TNF-α function [43], and activation of IL-1Ra and IL-10 [44]. Only few studies have addressed in vitro stimulation of immune cells by β2→1-fructans and cytokine profiles. In a study of Eiwegger *et al.* [45] three types of prebiotic oligosaccharides, including a mixture of short chain galactooligosaccharides (scGOS) and long chain β2→1-fructans were analysed for the induction of a selected panel of cytokines in cord blood mononuclear cells (CBMCs). The mixture containing long chain β2→1-fructans did not induce IL-10 in these experiments which corroborates our findings. Long chain β2→1-fructans alone, or short chain β2→1-fructans were not tested in the Eiwegger study, which would allow for a more complete comparison with our results. In supplementation studies in experimental animals, the observed immune effects with β2→1-fructans such as increased cytokine productions, increased serum and secretory immunoglobulins [13], [46]–[49], and increased numbers of IL-10^+^, TLR2^+^, and TLR4^+^ DCs [50] could be due to direct effects of β2→1-fructan-mediated TLR activation in the intestine, be it on immune cells or intestinal epithelial cells [51]. However, the underlying mechanisms in these in vivo experiments are likely to be more complex and are probably the result of combined direct and indirect effects in the intestine.

Also other studies corroborate our findings on chain length effects and differences in induced immune parameters. In a study where rats were supplemented with shorter chain β2→1-fructans (DP2-8), increased ex vivo secretions of IL-10 were observed in cells from the Peyer’s patches [13], [52]–[54] and mesenteric lymph nodes [53]. In another study by Ito *et al*. the effect of different chain length β2→1-fructans on prebiotic and immunomodulatory parameters in rats was studied [55]. In this study, β2→1-fructans supplementation increased the cecal lactobacilli and bifidobacteria counts and that IgA concentrations were increased in the order DP4> DP8> DP16. In addition, DP4, DP8, and DP16, but not DP23, increased IgA-producing plasma cells in the cecal mucosa. IFN-γ and IL-10 production in cecal CD4(+) T cells was enhanced solely by DP4. These results confirm that in vivo, chain length of β2→1-fructans is also of importance to the immunological response and the fact that these parameters were not correlated to any of the observed bacterial changes suggests that this is a direct effect on (immune) cells. This size exclusion effect for direct signalling of β2→1-fructans in vivo was previously suggested by Seifert and Watzl [8].

As there are many families of PRRs with typical carbohydrate binding properties and immunomodulatory capacities [56], we applied a strategy to target TLRs and NODs: as shown in this study, the activating capacity of β2→1-fructans on THP-1 cells was MyD88 dependent, and activation of TLR2 expressing HEK cells was strongly induced, which implies that signalling through TLRs, and specifically TLR2, is important in the immunomodulatory capacity of β2→1-fructans [11], [27], [28], [57]. Other PRRs, i.e. C-type Lectin-like receptors or RIG-like receptors do not signal via MyD88, but utilise other mechanisms and molecules to transduce their signal [58], [59].

When studying β2→1-fructan mediated activation of HEK cells expressing individual TLRs or NODS, we observed that NF-κB/AP-1 was strongly induced via activation of TLR2, and to a lesser extent by TLR4, 5, 7, 8, and NOD2. No activation was observed for TLR3, 9, or NOD1. TLR3 does not signal to NF-κB/AP-1 via MyD88 but through its adapter Toll/interleukin-1 receptor homology-domain-containing adapter-inducing interferon-β (TRIF) [60]. However, activation by β2→1-fructans was virtually absent in the THP-1 DefMyD cells, indicating the importance of MyD88-related TLRs and no involvement of TLR3.

TLR2 has many natural ligands of bacterial, fungal, viral, and even endogenous nature [61]. One of these is zymosan, a yeast PAMP consisting of β-glucans [62], which bear similarity to β2→1-fructans because both are prebiotic polysaccharides with β-glycosidic bonds. Although β-glucans are different molecules, it is tempting to speculate that the mechanism of TLR ligation by β2→1-fructans could be similar to that of β-glucans, but this requires further biochemical studies.

Another way of inducing different signals might be mechanistic differences in receptor interactions at the cellular surface. It is possible that the shorter chains only activate a few receptors at a time, and the receptors may be located relatively distant from each other, whereas the longer chain β2→1-fructans might show a property of clustering the relevant receptors on the membrane, thereby creating a molecular complex which enhances signal transduction or alters the downstream outcome. This clustering mechanism has been described for lipopolysaccharide (LPS), clustering substantial numbers of TLR4 [63] and may be a relevant mechanism for other TLRs as well.

To our best knowledge this is the first study addressing the direct mechanism behind the immunomodulating capacity of specific β2→1-fructans, with the aim to get more insight into the protective associations attributed to dietary fibers in epidemiological studies [4]. We demonstrated the principle that β2→1-fructans possess direct signalling capacity on human immune cells, mainly through TLR2. These results suggest that direct TLR2 signalling events on immune cells could be part of the mechanism by which IL-10 production is induced in *in vivo* β2→1-fructan supplementation studies. Also we show structure-function relationships *in vitro* for β2→1-fructans which illustrate that caution should be taken in ascribing beneficial health effects to families of molecules with similar structural features, as seemingly minor differences in chain length could induce opposite effects.
